# Market Share of US Catholic Hospitals and Associated Geographic Network Access to Reproductive Health Services

**DOI:** 10.1001/jamanetworkopen.2019.20053

**Published:** 2020-01-29

**Authors:** Coleman Drake, Marian Jarlenski, Yuehan Zhang, Daniel Polsky

**Affiliations:** 1 Department of Health Policy and Management, University of Pittsburgh Graduate School of Public Health, Pittsburgh, Pennsylvania; 2 Johns Hopkins Bloomberg School of Public Health, Baltimore, Maryland

## Abstract

**Importance:**

Access to reproductive health services is a public health goal. It is unknown how geographic and health plan network availability of Catholic and non-Catholic hospitals may be associated with access to reproductive health services in the United States.

**Objective:**

To characterize the market share of Catholic hospitals in the United States, both overall and within Marketplace health insurance plans’ hospital networks.

**Design, Setting, and Participants:**

This cross-sectional study of US counties used data on hospitals’ Catholic affiliation and discharges, hospital networks in Marketplace health insurance plans, and US Census population data to construct a national, county-level data set. The Catholic hospital market share overall in each county and in Marketplace plans’ hospital networks in each county were calculated. The study examined whether the Catholic hospital market share was different within Marketplace networks compared with the counties they served. Data analysis was conducted in May and June 2018.

**Main Outcomes and Measures:**

The overall Catholic hospital market share was calculated on the basis of the share of discharges in Catholic hospitals in a county compared with all hospital discharges. Overall market share was categorized as minimal (≤2%), low (>2% to ≤20%), high (>20% to ≤70%), or dominant (>70%). The Catholic hospital market share in Marketplace networks was calculated as the share of Catholic hospital discharges in each Marketplace network.

**Results:**

The sample included 4450 hospitals in 3101 counties. Overall, 26.1% of US counties had minimal Catholic hospital market share, 38.6% had low Catholic hospital market share, and 35.3% had high or dominant Catholic hospital market share; 38.7% of US reproductive-aged women resided in counties with high or dominant Catholic hospital market share. Among counties with Catholic hospital market share greater than 2%, the distribution of the median Marketplace network’s Catholic hospital market share (median [interquartile range], 4.6% [0%-24.3%]) was lower than overall Catholic hospital market share (median [interquartile range], 18.5% [8.1%-36.5%]). The median Marketplace hospital network had a lower Catholic hospital market share than the county overall in 68.0% of US counties with Catholic hospital market share greater than 2%.

**Conclusions and Relevance:**

In this national study, 35.3% of counties had high or dominant Catholic hospital market share serving an estimated 38.7% of US women of reproductive age. Marketplace health insurance plans’ hospital networks included a lower share of Catholic hospitals than the counties they serve.

## Key Points

**Question:**

Is access to reproductive health services in the United States associated with the market share of Catholic hospitals?

**Findings:**

In this cross-sectional study of all US counties, 35.3% of US counties, where 38.7% of US women of reproductive age live, have a high Catholic hospital market share. Hospital networks in Marketplace health insurance plans included a lower share of Catholic hospitals than the overall county share.

**Meaning:**

Restricted access to reproductive health services associated with high Catholic hospital market share is common in US counties, but health insurance plans’ hospital networks do not exacerbate this issue.

## Teaser

This cross-sectional study examines whether access to reproductive health services in the United States is associated with the market share of Catholic hospitals at the county level.

## Introduction

Access to safe and effective reproductive health services is widely recognized as a public health policy goal.^1^ The Patient Protection and Affordable Care Act contained several provisions to expand access to reproductive health services. These included expanding insurance coverage to an estimated 20 million people, requiring insurers to cover maternity care, and requiring insurers to cover the full range of US Food and Drug Administration–approved contraceptive methods without patient cost-sharing.^2,3,4^ Rigorous empirical work suggests that these provisions have contributed to better access to contraceptive care and may have improved pregnancy-related outcomes.^5,6,7,8^

A critical barrier to access to reproductive health services may be directives from religious health care organizations.^9,10,11,12^ In particular, Catholic hospital systems follow a religious directive that prohibits the provision of contraception, infertility treatments, sterilization for contraceptive purposes, and induced abortion.^13^ If prospective patients were both aware of these restrictions and had unimpeded access to health systems offering the care restricted by Catholic hospital systems, the ultimate impact on health would be limited. However, a recent survey^14^ exploring the issue of awareness found that 37% of women whose primary hospital system was Catholic were unaware of that fact. This study explores factors that may impede health system access.

Geographic availability of health care systems is a significant determinant of access to care.^15,16^ We posit that there are 2 ways that the geography of available Catholic and non-Catholic hospital systems might be associated with access to reproductive health care services. The first way is based on the concentration of the supply of Catholic hospital systems. In geographic areas where Catholic hospital systems dominate the market, these systems are difficult to avoid. In areas where they have no presence, Catholic hospital restrictions do not play a role at all. Given this supply, health insurance can play a strong role in driving demand via hospital networks—that is, the set of hospitals available to enrollees in a given health plan. A health plan’s hospital network essentially constrains the choice of hospitals among beneficiaries to those hospitals that are in the plan’s network. Thus, both the concentration of Catholic hospitals in a geographic area and the degree to which health plan hospital networks disproportionately exclude or include Catholic hospitals work together to affect the probability that women receive care in a Catholic hospital that does not provide many reproductive health services.

In this study, we sought to determine how access to reproductive health services might be shaped by the availability of Catholic and non-Catholic hospitals in the United States. First, we measured the market share of Catholic hospitals in each county according to the percentage of discharges from Catholic hospitals among county residents. Second, we measured how access to reproductive health services might be altered by health plans’ in-network hospitals on the basis of Marketplace insurance plans’ hospital networks. The Marketplaces were created by the Patient Protection and Affordable Care Act to provide health insurance coverage to nonelderly, nondisabled adults who do not have affordable offers of health insurance via an employer and do not qualify for Medicaid. In 2016, 6.8 million women enrolled in Marketplace plans.^17^ Given that the Marketplaces primarily insure nonelderly adults, they are an important source of insurance coverage for women of reproductive age. Marketplace plans’ networks tend to be narrow.^18^ They may, thus, direct women away from or toward hospitals providing reproductive health services by covering a disproportionate share of Catholic or non-Catholic hospitals. The Marketplaces also are of particular interest because Marketplace insurers’ networks have received little regulatory scrutiny since their launch in 2014. As such, they provide a clear picture of how insurers without regulatory oversight may promote or decrease access to reproductive health services. We tested differences in counties’ Catholic hospital market shares overall and in their Marketplace insurance plans’ networks to assess whether Marketplace plans provide greater access to reproductive health services compared with the counties they serve. Finally, we quantified the share of counties in which Marketplace plans’ networks provided greater access to reproductive health services compared with counties they serve.

## Methods

### Overview

Because this is a cross-sectional study of hospital market share and insurance networks, not of living individuals, it does not constitute human subjects research. Institutional review board approval thus was not required, in accordance with 45 CFR §46.102(f). This study follows the Strengthening the Reporting of Observational Studies in Epidemiology (STROBE) reporting guideline.

We analyzed the Catholic hospital market shares of each county in the continental United States. We then examined differences in the demographic characteristics and Catholic hospital market shares of Marketplace insurance networks of counties according to whether they had high or low Catholic hospital market share.

### Data Sources

Our study included 3 main data sources to create county-level measures of Catholic hospital market share overall and in health insurance hospital networks in 2016, the most recent year of data available. First, to identify hospital characteristics, we used the American Hospital Association (AHA) Annual Survey Database from 2015,^19^ the most recent year of data available at the time of our analysis. The AHA survey data are completed annually by hospital administrators and represent a comprehensive survey of US hospitals. We used the AHA data to identify the location and Catholic affiliation of each general acute care hospital, according to self-identified Catholic hospital status. The 2015 AHA survey response rate was 83%.^19^ The AHA used its internal database to populate values for nonrespondents for the variables we used in our analysis: hospital location, Catholic affiliation, and hospital type.

Second, to measure hospital market share according to the share of discharges, we used the Centers for Medicare & Medicaid Services’ Medicare Provider Analysis and Review (MedPAR) data.^20^ The MedPAR data include a census of all hospital Medicare hospital discharges by hospital and patients’ home zip codes. We used MedPAR data to identify each hospital’s market share in each zip code. Although women of reproductive age generally are not Medicare beneficiaries, using state inpatient discharge data, we found market share based on the Medicare discharge data to be representative of all discharges, not just those for Medicare beneficiaries. This is because a hospital’s volume of Medicare patients in a given area is associated with its total volume of patients.

Third, to determine hospitals’ inclusion in particular health insurance plans’ networks, we used network data from Vericred.^21^ Vericred is a health care data services company that collects data on the health plans offered in each state’s Health Insurance Marketplace, including data on the networks of hospitals offered by each plan. We used Vericred data to identify the set of networks available to Marketplace enrollees by county and the hospitals included in each of these networks. The Vericred data contain information on 476 Marketplace hospital networks. Hospital network data were missing for the remaining 111 networks, which we dropped from our analysis. We also obtained county-level demographic information from the 2016 American Community Survey,^22^ county-level religious affiliation data from the 2010 US Religion Census^23^—the most recent year of data available—and county rurality designations used by the Centers for Medicare & Medicaid Services for network adequacy regulation for Medicare Advantage plans.

We excluded Alaska and Hawaii from our county analysis because of the unique geographies of these states, and Loving County, Texas, because of its small female population aged 15 to 44 years; thus, our final analytic sample included 3101 of the 3102 counties in the continental United States. Among the 587 insurer networks available in the Marketplaces in 2016, hospital networks were available for 476 (81%) of networks in the Vericred data.^21^ County-level analysis of Marketplace networks includes 3079 of the 3101 counties. We excluded 22 counties because data on Marketplace networks lacked nearby in-network hospitals and/or MedPAR discharges corresponding to nearby hospitals.

### Measures

#### Catholic Hospital Market Share Overall

We measured Catholic hospitals’ overall market share in each county. To do so, we first identified Catholic and non-Catholic hospitals using the AHA data. From the count of discharges for each hospital–zip code dyad provided by the MedPAR data, we first excluded hospitals that are more than 50 miles from the zip code’s geographic center and that have less than 0.5% of the zip code’s discharges. Catholic hospital market share at the zip code level is simply the share of discharges from Catholic hospitals among residents of the zip code. Next, we aggregated Catholic hospital market shares from the zip code to the county level using a weighted average of hospital market shares among zip codes in each county. The zip codes were weighted by their female population aged 15 to 44 years (ie, reproductive-aged women) as identified in the 5-year 2016 American Community Survey. The zip codes crossing county lines were assigned to the county where most of their population resided. We grouped counties by their level of Catholic hospital market share: minimal (≤2%), low (>2% to ≤20%), high (>20% to ≤70%), and dominant (>70%).

#### Catholic Hospital Market Share in Marketplace Networks

We then measured, for each Marketplace health insurance network in each county where a plan using that network is sold, the Catholic hospitals’ market share among the in-network hospitals. Because the number of Marketplace plan networks per county ranged from 1 to 19, with a mean of approximately 4, this measure was estimated for 12 838 network-county dyads in the continental United States. We identified hospitals’ participation in networks with Vericred data.^21^

Because the Vericred data define hospitals using National Provider Identifiers and a single hospital can be assigned multiple National Provider Identifiers, we consolidated National Provider Identifiers sharing the same AHA identifier.^24^ Because enrollees typically have a choice of networks within the county where they reside, this analysis considers the implications across the range of options. Our primary measure uses the median Marketplace network, defined as the network in a given county with the median Catholic hospital market share among networks. This was our primary measure because it is unlikely that enrollees are selecting plans according to in-network hospitals’ religious affiliations.^14^ This measure thus captures a typical network in the market. We considered 2 alternative measures to quantify access to reproductive health services faced by enrollees in extreme scenarios. For the first alternative measure, we calculated the Catholic hospital market share faced by an enrollee who deliberately selects the plan with the lowest Catholic hospital market share (ie, the least Catholic network). For the second alternative measure, we calculated the Catholic hospital market share faced by an enrollee who, perhaps unaware, selects the plan with the lowest access to reproductive health services (ie, the most Catholic Marketplace network). These measures summarize geographic access to reproductive health services for a woman selecting the network that maximizes geographic access (least Catholic), the standard or normal network (median), and the network that minimizes geographic access (most Catholic).

### Statistical Analysis

We used 2-tailed tests of statistical significance to test whether the characteristics of residents in the counties with low reliance on Catholic hospitals for inpatient services were different from those of residents in the counties with high or dominant Catholic hospital market share. We used the Wilcoxon signed-rank test to test whether each characterization of Catholic hospital market share (ie, least, median, and most Catholic) is different within Marketplace networks compared with the county hospital supply as a whole. The Wilcoxon signed-rank test determines whether we should reject the null hypothesis that the distribution of Catholic hospital market share in counties overall and in Marketplace plan networks is the same (ie, Marketplace plan networks do not change women’s access to reproductive health services).

We also used 2-tailed tests of significance to test for differences in the percentages of counties where Marketplace networks provide greater access to reproductive services, compared with the county as a whole, between counties with high or dominant and low Catholic market shares. Because Marketplace networks might have a greater association with access to reproductive health services in counties where Catholic hospitals are dominant, we summarize these percentages separately in counties with high or dominant market shares and those with low market shares. Counties with a minimal market share of Catholic hospitals were excluded from this analysis, because it is not feasible for a Marketplace network to restrict access to reproductive services in such counties.

We conducted all analyses in May and June 2018. Analyses were conducted using Stata statistical software version SE 14.2 (StataCorp) and SAS statistical software version 9.4 (SAS Institute). Results from all statistical tests were reported with *P* < .05 considered statistically significant.

## Results

**Figure 1.**
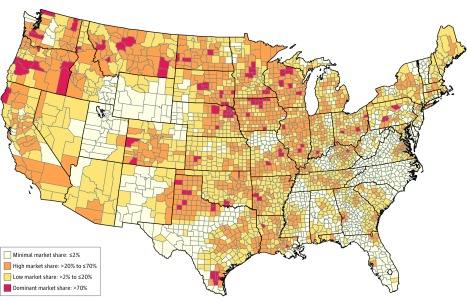
Catholic Hospital Market Share of US Counties Map shows the overall Catholic hospital market shares in all continental US counties. Counties are shown by their Catholic hospital market shares (minimal, low, high, or dominant).

The sample included 4450 hospitals in 3101 counties. Figure 1 quantifies the overall Catholic hospital market shares in all continental US counties. Counties are shown by their Catholic hospital market shares (minimal, low, high, or dominant). There was large variation across counties in Catholic hospital market shares: 993 counties had a high market share (>20% to ≤70%) and 101 counties had a dominant market share (>70%). Although the mean Catholic hospital market share was 18.4%, 35.3% of counties had a market share greater than 20%, 26.1% of US counties had a minimal Catholic hospital market share, and 38.6% had a low Catholic hospital market share. Counties with high and dominant Catholic market shares tended to be located on the West Coast, the Midwest (particularly the upper Midwest), the eastern megalopolis stretching from Boston to New York, and the south-central United States, including urban areas in Texas, Colorado, and Oklahoma. The South that is east of the Mississippi and the Mountain West tended to have low or minimal Catholic hospital market shares.

There were small differences in Catholic hospital market share by some demographic characteristics (Table 1). Among the population of women of reproductive age, 38.7% lived in counties with high or dominant Catholic hospital market share, and 35.4% and 25.9% lived in counties with low and minimal market shares, respectively. The mean (SD) proportion of the population who identify as Catholic was 15.8% (13.8%) in counties with high or dominant Catholic hospital market share, compared with 12.6% (13.5%) in counties with low Catholic market share (difference, 3.2%; 95% CI, 2.0% to 4.3%; *P* < .001). Populations in counties with high or dominant Catholic hospital market share were more likely to be white (mean [SD], 86.7% [13.5%] vs 83.4% [17.5%]; difference, 3.2%; 95% CI, 1.9% to 4.5%; *P* < .001), Hispanic (mean [SD], 8.9% [13.5%] vs 8.0% [11.6%]; difference, 0.9%; 95% CI, −0.1% to 1.9%; *P* = .08), more educated (ie, some college or more; mean [SD] 52.6% [10.0%] vs 50.5% [10.7%]; difference, 2.1%; 95% CI, 1.3% to 3.0%; *P* < .001), and have higher annual median incomes (mean [SD], $49 400 [$11 000] vs $47 300 [$13 100]; difference, $2100; 95% CI, $1100 to $3100; *P* < .001) than their counterparts in counties with lower Catholic hospital market share. The magnitudes of these differences, however, were small. Large differences exist, however, by Census regions. Nearly one-half (49.2%) of counties with high or dominant Catholic hospital market share were in the Midwest, whereas 35.6% of counties with low Catholic hospital market share were Midwestern. Southern counties, on the other hand, had disproportionately low Catholic hospital market shares (high or dominant market share, 31% vs low market share, 42.5%).

**Table 1.** County Demographic Characteristics by Catholic Hospital Market Share^a^

Characteristic	Overall	Market Share		Difference (95% CI)	*P* Value^b^
		Low	High or Dominant		
County characteristics					
Counties, No.	2291	1197	1094	NA	NA
Female population of reproductive age (millions), No.	464	222	243	NA	NA
Female population of reproductive age, % of national total	74.1	35.4	38.7	NA	NA
Demographic characteristics					
Female, mean (SD), %	49.9 (2.3)	49.9 (2.5)	49.8 (2.0)	−0.1 (−0.3 to 0.1)	.46
Female of reproductive age (15-44 y), mean (SD), %	17.2 (2.8)	17.3 (2.8)	17.2 (2.7)	−0.0 (−0.3 to 0.2)	.81
Race, mean (SD), %					
White	85.0 (15.8)	83.4 (17.5)	86.7 (13.5)	3.2 (1.9 to 4.5)	<.001
Black	7.7 (13.6)	9.3 (15.6)	6.0 (10.9)	−3.3 (−4.4 to −2.2)	<.001
Asian	1.2 (2.4)	1.2 (2.4)	1.2 (2.3)	0.0 (−0.2 to 0.2)	.92
Other	6.1 (8.6)	6.1 (9.4)	6.2 (7.7)	0.1 (−0.6 to 0.8)	.78
Hispanic ethnicity, mean (SD), %	8.4 (12.5)	8.0 (11.6)	8.9 (13.5)	0.9 (−0.1 to 1.9)	.08
Education, some college or more, mean (SD), %	51.5 (10.4)	50.5 (10.7)	52.6 (10.0)	2.1 (1.3 to 3.0)	<.001
Annual household income, median, mean (SD), $US in thousands	48.3 (12.1)	47.3 (13.1)	49.4 (11.0)	2.1 (1.1 to 3.1)	<.001
Rural county, No. (%)	1772 (77.3)	939 (78.4)	833 (76.1)	NA	.19
Census regions, No. (%)					
Northeast	154 (6.7)	113 (9.4)	41 (3.7)	NA	<.001
Midwest	964 (42.1)	426 (35.6)	538 (49.2)	NA	
South	850 (37.1)	509 (42.5)	341 (31.2)	NA	
West	323 (14.1)	149 (12.4)	174 (15.9)	NA	
Catholic population, mean (SD), %	14.1 (13.8)	12.6 (13.5)	15.8 (13.8)	3.2 (2.0 to 4.3)	<.001

Abbreviation: NA, not applicable.

^a^ The unit of observation was the county. County characteristics were obtained from the 5-year 2016 American Community Survey,^22^ except the percentage of Catholic individuals, which was obtained from the 2010 US Religion Census (179 missing).^23^ County rurality was based on the Centers for Medicare & Medicaid Services definitions, and rural counties include 3 county type designations: micro, rural, and counties with extreme access considerations. The table excludes 810 counties with minimal (≤2%) market share of Catholic hospital.

^b^ *P* values test the difference in means between counties with low (>2% to ≤20%) and high or dominant (>20%) Catholic hospital market shares for continuous variables, with the χ^2^ test used for categorical variables.

**Figure 2.**
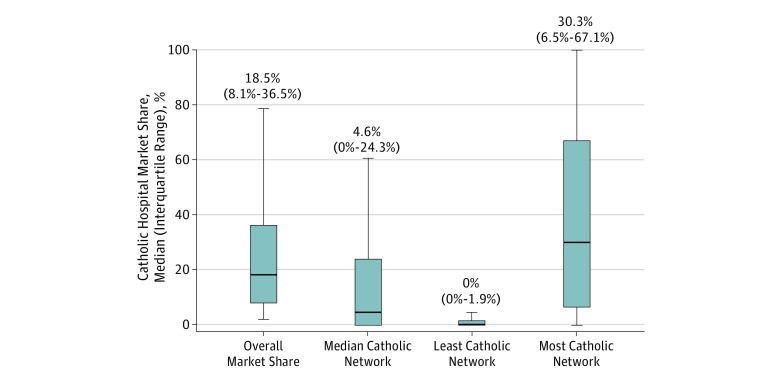
Distributions of Catholic Hospital Market Shares Within Marketplace Networks by Catholic Hospital Market Share The distribution of the median Marketplace network’s Catholic hospital market share (median [interquartile range {IQR}], 4.6% [0%-24.3%]) is lower than the overall Catholic hospital market share (median [IQR], 18.5% [8.1%-36.5%]). The median (IQR) market share for the least Catholic network is 0.0% (0.0%-1.9%) and that for the most Catholic network is 30.3% (6.5%-67.1%). The figure excludes 820 counties with minimal (≤2%) market share of Catholic hospitals or counties where available Marketplace networks did not accurately represent the Marketplace. Lines within boxes show medians, tops and bottoms of boxes show 75th and 25th percentiles, respectively, and error bars show 95% CIs.

Figure 2 shows the distributions of the median, least, and most Catholic Marketplace networks’ Catholic hospital market shares compared with overall Catholic hospital market share among counties with Catholic hospital market share greater than 2%. The distribution of the median Marketplace network’s Catholic hospital market share (median [interquartile range], 4.6% [0%-24.3%]) was lower than overall Catholic hospital market share (median [interquartile range], 18.5% [8.1%-36.5%]). The distributions of the least and most Catholic Marketplace networks were lower and higher than overall Catholic hospital market share, respectively. Table 2 shows the differences between Marketplace networks’ Catholic hospital representation compared with the county as a whole. We found that 1552 counties (68.0%) had greater access to reproductive health services in the median Marketplace network and 2092 counties (91.7%) had greater access to reproductive health services in the least Catholic Marketplace network. As expected, this fraction was less than 50% in the most Catholic network, but these extreme networks still provided greater access to reproductive health services in 642 counties (28.2%). This table also estimates this summary measure separately among counties with low market shares and those with high or dominant market shares. Although the percentage of counties in each subgroup was similar, for the median case in the high or dominant group of counties compared with the low group (70.3% vs 65.6%), it was 4.7 percentage points less (95% CI, −8.5% to −0.9%; *P* = .02).

**Table 2.** Percentage of US Counties Where Marketplace Hospital Networks Provide Greater Access to Reproductive Health Services^a^

County Marketplace Network	Counties, No. (%)			Difference (95% CI)	*P* Value^b^
	All (n = 2281)	Market Share			
		Low (n = 1188)	High or Dominant (n = 1093)		
Least Catholic	2092 (91.7)	1093 (92.0)	999 (91.4)	−0.6 (−2.9 to 1.7)	.60
Median	1552 (68.0)	835 (70.3)	717 (65.6)	−4.7 (−8.5 to −0.9)	.02
Most Catholic	642 (28.2)	364 (30.6)	278 (25.4)	−5.2 (−8.9 to −1.5)	.01

^a^ A network is defined as providing greater access to reproductive health services when its Catholic hospital market share is smaller than or equal to the overall Catholic hospital market share of the county in which it operates. The table excludes 820 counties with minimal (≤2%) market share of Catholic hospital or counties where available Marketplace networks did not accurately represent the Marketplace.

^b^ *P* values test the difference in means between counties with low (>2% to ≤20%) and high or dominant (>20%) Catholic hospital market shares.

**Figure 3.**
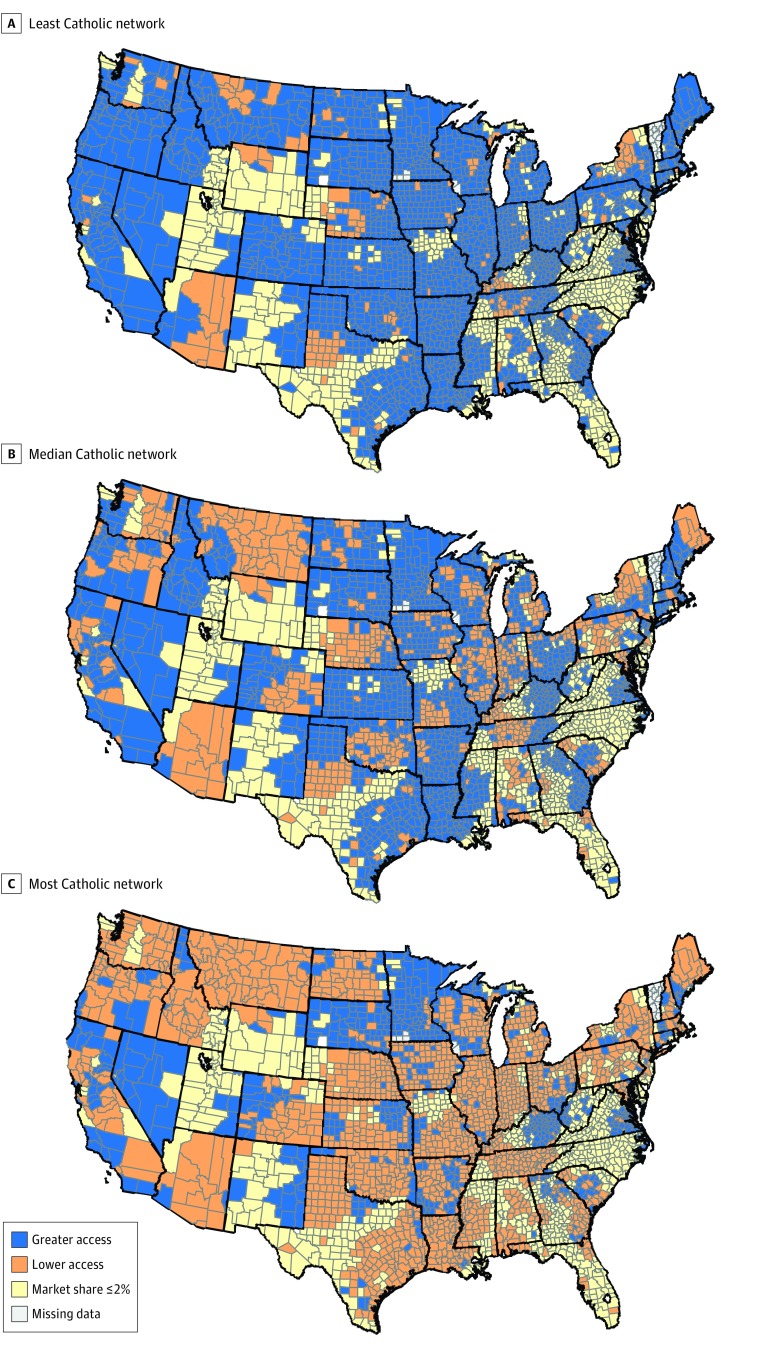
Counties Where Marketplace Networks Provide Greater Access to Reproductive Health Services Map shows counties according to access to reproductive services and whether their least (A), median (B), and most (C) Catholic Marketplace networks have lower Catholic market shares than their respective counties overall.

Figure 3 classifies counties according to whether their least, median, and most Catholic Marketplace networks had lower Catholic market shares than their respective counties overall. With the exception of 189 counties (8.3%) (eg, upstate New York and Arizona), it was possible for women to select a Marketplace network that is less Catholic than the county overall. However, it also is possible for women to select a Marketplace network with greater than 80% Catholic hospital market share in 440 counties (19.3%). This occurs primarily in counties with higher Catholic hospital market share, which often have at least 1 Marketplace hospital network dominated by Catholic hospitals.

## Discussion

In US counties, the median Catholic hospital market share was 18.5%, but there was large variation that includes 993 counties with high market shares (>20% to ≤70%) and 101 counties with dominant market shares (>70%). Although the mean Catholic hospital market share was 18.4%, 35.3% of counties have market shares greater than 20%. These counties at the high and dominant level, which are the counties at greater risk of reduced access to reproductive health services, were home to 38.7% of US women of reproductive age. These findings suggest that Catholic hospital reproductive health policies are associated with access to reproductive health services for a substantial fraction of women who may require these services.

We also found that the Catholic hospital market shares in Marketplace health insurance networks were lower than the counties they served. The median Marketplace network included a smaller share of Catholic hospitals, compared with the overall county Catholic hospital market share, in 68.0% of counties. These findings suggest that Marketplace health insurance networks, on average, provide greater access to reproductive health services, compared with the counties they serve. Most counties with high or dominant Catholic hospital market shares were in the upper Midwest, spreading across the Pacific Northwest. Notably, we did not observe large differences between the percentage of the population who are Catholic by the market share of Catholic hospitals.

These findings suggest wide geographic variability in terms of whether the Catholic hospital market share might pose a barrier to obtaining reproductive health care. This is concerning given that the United States consistently ranks poorly compared with other wealthy nations on measures of women’s reproductive health and has an unacceptably high rate of maternal mortality.^25^ An estimated 10% of US women have unmet needs for family planning services, although these unmet needs are substantially greater for socially disadvantaged groups.^26^ Little research has examined the implications of the Catholic health care system market share on health outcomes, although 1 study^27^ found that hospital affiliation with a Catholic health care system reduced tubal ligation rates by 30%. Other studies^28,29,30^ investigating the effects of structural changes on the health care system suggest that reduced access to reproductive health services may be adversely associated with health outcomes. For example, prior work has found that hospital obstetric unit closures in rural areas are associated with increased risk of preterm birth^29^ and that restricting access to Planned Parenthood clinics is associated with a decrease in contraceptive use and a concurrent increase in maternal mortality.^29,30^

Concerns about restricted access to reproductive health services could be mitigated if patients were able to accurately choose hospitals whose services meet their needs and values.^31^ Even in circumstances in which women have a choice between Catholic and non-Catholic hospitals, it may not be a simple matter to determine the reproductive services offered by available hospitals. Health insurance networks themselves may be opaque at the time when women enroll.^32^ In addition, most Catholic hospitals’ websites portray their affiliation as part of a larger secular health care system, and only 28% of Catholic hospitals disclose on their websites which services are not available because of religious directives.^33^ When women request family planning appointments, Catholic health care centers often do not specify which reproductive services are banned.^34^

State-based health insurance Marketplaces were created under the Affordable Care Act to improve health insurance coverage and access to care for nonelderly adults, and policy makers explicitly mandated that insurance cover both maternity and contraceptive services.^35^ However, currently there are no oversight standards with respect to realized access to reproductive services.^36^ There is a high amount of heterogeneity by geography with little association with population characteristics. Hospital network adequacy standards exist and could be helpful for network-related limited access,^37^ but they currently do not consider whether hospitals are Catholic. Adding this metric to network adequacy standards could be one policy option to monitor access to reproductive services in the Marketplaces.

### Strengths and Limitations

Major strengths of this study include nationally representative data and our ability to quantify how health insurance networks are associated with the market share of Catholic hospitals faced by patients. This study also has limitations. First, the use of Marketplace network data precluded us from generalizing our findings about Catholic hospital market share to other insurance networks, such as Medicaid managed care plans. This is an important limitation given that state Medicaid programs are the largest single payer for health care for nonelderly women. Medicaid managed care networks also may have charitable objectives in line with Catholic hospitals, which may mean that Medicaid networks are relatively more Catholic than their Marketplace counterparts. We note that the overall estimates of Catholic hospital market share are national in scope. Second, we are unable to measure whether Catholic hospitals provide systems whereby patients could be referred for reproductive health services elsewhere. Even a referral system for reproductive health services would likely still pose a barrier to care for many patients, however. Third, our study examined the market share of Catholic hospitals, as opposed to the market share of Catholic health care systems. Therefore, these findings are informative to understand geographic variability in access to reproductive health services occurring in inpatient settings or hospital-owned outpatient clinics, but do not necessarily generalize to access to outpatient services.

## Conclusions

This is the first study, to our knowledge, to provide national estimates of Catholic hospital market share in the United States. We found that 35.3% of US counties, where 38.7% of US women of reproductive age live, had a high Catholic hospital market share. Marketplace health insurance networks tend to include a lower share of Catholic hospitals than the overall county share, suggesting that Marketplace networks are protective of access to reproductive health services. The geographic variation in availability of Catholic and non-Catholic hospital systems is an important factor for consideration in the study of reproductive health outcomes in the United States.
